# Difference in the early postoperative change of the joint line convergence angle between opening wedge and closed wedge high tibial osteotomies

**DOI:** 10.1186/s13018-021-02214-x

**Published:** 2021-01-19

**Authors:** Ken Kumagai, Hiroshi Fujimaki, Shunsuke Yamada, Shuntaro Nejima, Joji Matsubara, Yutaka Inaba

**Affiliations:** Department of Orthopaedic Surgery, Yokohama City University Hospital, 3-9 Fukuura, Kanazawa-ku, Yokohama, 236-0004 Japan

**Keywords:** High tibial osteotomy, Opening wedge, Closed wedge, Knee, Alignment, Joint line convergence angle

## Abstract

**Background:**

The purpose of this study was to investigate the correction error associated with soft tissue balance in high tibial osteotomy (HTO) and the difference between opening wedge HTO (OWHTO) and closed wedge HTO (CWHTO).

**Methods:**

A total of 170 knees of 130 patients (85 knees of 68 patients in OWHTO and 85 knees of 62 patients in CWHTO) were evaluated. Anteroposterior radiographs of the knee and full-length leg were taken preoperatively, immediately under general anesthesia postoperatively, 2 days, and 1 and 12 months postoperatively. The femorotibial angle (FTA), joint line convergence angle (JLCA), and medial proximal tibial angle (MPTA) were measured.

**Results:**

The postoperative FTA was decreased from 170.5 ± 2.1° at 0 day to 168.6 ± 2.2° at 2 days in OWHTO (*P* < 0.05), whereas it was not changed from 168.7 ± 2.4° at 0 day to 168.1 ± 2.8° at 2 days in CWHTO. The JLCA was 4.8 ± 1.8° preoperatively, 4.2 ± 1.9° at 0 day, 2.2 ± 1.8° at 2 days (*P* < 0.05 vs 0 day), 2.6 ± 1.7° at 1 month, and 2.7 ± 1.6° at 12 months in OWHTO, and 7.1 ± 3.2° preoperatively, 4.1 ± 2.4° at 0 day (*P* < 0.05 vs preoperative), 3.4 ± 2.5° at 2 days, 3.9 ± 2.3° at 1 month, and 4.2 ± 2.6° at 12 months in CWHTO. Multiple regression analysis showed that preoperative factors affecting change of the JLCA from preoperative to postoperative 1 month were the correction angle in OWHTO (*P* = 0.001) and the preoperative standing JLCA in OWHTO (*P* < 0.001) and CWHTO (*P* < 0.001).

**Conclusions:**

A significant decrease of the JLCA occurred immediately after osteotomy under anesthesia in CWHTO, whereas in OWHTO there was no decrease under anesthesia, but it decreased several days postoperatively.

## Introduction

High tibial osteotomy (HTO) is an established realignment procedure to alter the mechanical load for medial compartmental osteoarthritis (OA) of the knee. Accurate correction is mandatory for success of the surgical procedure in HTO, since the long-term clinical outcomes depend on postoperative lower limb alignment [1, 2]. Various computer-aided tools including preoperative digital planning software and intraoperative navigation systems have recently been developed to perform accurate correction [3, 4]. However, accurate intraoperative correction is often lost after HTO due to changes of soft tissue balance as indicated by the joint line convergence angle (JLCA) [5].

Correction error associated with changes of the JLCA is thought to occur due to a discrepancy between intraoperative and postoperative conditions in the assessment of limb alignment. Preoperative planning is usually carried out using standing radiographs. In contrast, intraoperative assessment of lower limb alignment is performed under non-weight-bearing conditions with general anesthesia. Therefore, it is difficult to predict the effect of soft tissue balance on postoperative limb alignment under different conditions. Filling the gap between preoperative or intraoperative prediction and the postoperative real measurements is a key to success in the achievement of more accurate correction.

Recent studies showed that one of the factors associated with correction error is collateral laxity in both opening wedge HTO (OWHTO) [6, 7] and closed wedge HTO (CWHTO) [8]. In addition, to avoid unexpected correction error, predictive methods including use of supine radiographs [9, 10], a predictive formula [11, 12], or use of standing radiographs on a lateral wedge insole [13] have been introduced. However, differences including the frequency, extent, and timing of the occurrence of correction error associated with soft tissue balance between OWHTO and CWHTO have not been well documented, and these differences between the two HTO procedures need to be elucidated to achieve ideal postoperative alignment.

The purpose of this study was to investigate the difference in correction error associated with soft tissue balance between OWHTO and CWHTO. It was hypothesized that early postoperative change of the JLCA is found in both HTO procedures, but its extent and timing differ between the two HTO procedures.

## Materials and methods

### Patients

A total of 170 knees of 130 patients with medial compartmental OA of the knee who underwent HTO were investigated. The inclusion criterion was painful OA localized to the medial compartment of the knee. The exclusion criteria were OA of the lateral compartment, flexion contracture > 15°, or a history of osteonecrosis of the knee, inflammatory arthritis, joint infection, or immunosuppressive therapy. The decision for either technique was made preoperatively according to the correction angle based on our institutional protocol [14]. OWHTO was performed in 85 knees of 68 patients with a correction angle ≤ 15°, and CWHTO was performed in 85 knees of 62 patients with a correction angle > 15°. Demographic data are shown in Table 1. This study was approved by the institutional review board at our hospital (#B180200061).

**Table 1:** Demographic data

	OWHTO	CWHTO
Number of patients (knees)	68 (85)	62 (85)
Male/female	16 (20)/52 (65)	23 (29)/39 (56)
Age (years)	64.1 ± 8.4	64.3 ± 12.5
Body mass index (kg/m^2^)	24.5 ± 3.2	25.3 ± 3.1
OA grade* 2/3/4 (knees)	57/27/1	13/46/26

*OA grade modified from Ahlbach’s classification

### Surgical procedure and postoperative management

The amount of angular correction was planned preoperatively with the aim of achieving tibiofemoral anatomical valgus of 10° in a one-leg standing radiograph postoperatively.

OWHTO was performed using an anteromedial approach under fluoroscopic guidance. The osteotomy was started 35 mm below the medial articular surface of the tibia. An oblique osteotomy was performed from the medial cortex to the upper third of the proximal tibiofibular joint using biplanar technique, leaving the tibial tuberosity intact. The osteotomized gap was gradually opened and filled with two wedged blocks of β-TCP with 60% porosity (Osferion, Olympus Terumo Biomaterials. Corp., Tokyo, Japan) and fixed with TomoFix (DePuy Synthes, Zuchwil, Switzerland).

CWHTO was performed using an anterolateral approach under fluoroscopic guidance after fibular osteotomy. The osteotomy was started 30 mm below the lateral articular surface of the tibia. The proximal osteotomy was performed parallel to the tibial plateau, and the distal osteotomy was performed obliquely toward the hinge point at 1 cm from the medial cortex, with a flange to leave the insertion of the patellar tendon with a distal fragment. The osteotomy gap was closed and fixed with OWL plate (Mizuho Ikakogyo Co., Ltd., Tokyo, Japan) on the lateral side and TomoFix on the medial side.

Patients started a postoperative rehabilitation program including isometric quadriceps and range-of-motion exercises 1 day postoperatively. In CWHTO, a non-weight-bearing regimen was prescribed for 2 weeks, followed by partial weight-bearing exercise, and full weight-bearing exercise was permitted 3 weeks postoperatively. In OWHTO, a non-weight-bearing regimen was prescribed for 1 week, followed by full weight-bearing exercise. Casts or supportive devices were never applied in both procedures.

### Assessment of radiographic outcomes

For radiographic assessment, anteroposterior radiographs of the knee were taken in the standing position preoperatively and 1 and 12 months postoperatively, and in the supine position immediately after surgery under general anesthesia and 2 days postoperatively. Limb alignment is expressed as the femorotibial angle (FTA), measuring the lateral angle between the anatomical femoral and tibial axes [2, 15]. The JLCA was measured as the angle formed between a line tangent to the distal femoral condyle and the proximal tibial plateau [6]. Full-length anteroposterior radiographs of the lower limb were taken in the standing position preoperatively and 1 and 12 months postoperatively. The medial proximal tibial angle (MPTA) was measured as the medial angle formed between the tibial mechanical axis and the knee joint line of the tibia [16].

### Statistical analysis

Statistical analysis was carried out using BellCurve for Excel version 2.21 (Social Survey Research Information, Tokyo, Japan). The Mann-Whitney *U* test and the Kruskal-Wallis test were used to analyze radiographic outcomes between preoperative and postoperative measurements, or two HTO procedures. Pearson’s chi-squared test was used to test for significant differences in the distributions of change of the JLCA. Pearson’s correlation coefficient was used to identify relationships between change of the JLCA and each preoperative factor. Multiple regression analysis was performed to identify factors affecting change of the JLCA. An adjusted *P* value < 0.05 was considered significant. A power calculation showed that a sample size of 86 in each osteotomy procedure could detect differences with an effect size of 0.5, with 5% probability of a type I error and power of 90%. Thus, the required sample was determined to be 85 knees in each group. The intra-rater and inter-rater reliabilities of radiographic measurements were assessed by calculating intraclass correlation coefficients (ICCs).

## Results

### Postoperative changes in FTA, JLCA, and MPTA

Preoperative and postoperative measurements of the FTA, JLCA, and MPTA are summarized in Table 2. There were significant differences in the preoperative standing FTA, JLCA, and MPTA between OWHTO and CWHTO (*P* < 0.05). The FTA and JLCA were decreased postoperatively in both HTO procedures (*P* < 0.05). The postoperative FTA was significantly different between the two HTO procedures on the operation day (*P* < 0.05), but it was not significantly different thereafter. The postoperative FTA was significantly decreased from 0 day to 2 days in OWHTO (*P* < 0.05), whereas it was not significantly altered during the postoperative period in CWHTO. Similarly, the postoperative JLCA was significantly decreased from 0 day to 2 day in OWHTO (*P* < 0.05), whereas it was not significantly altered during the postoperative period in CWHTO. The MPTA was increased postoperatively in both HTO procedures (*P* < 0.05). The postoperative MPTA was greater in CWHTO than in OWHTO (*P* < 0.05).

**Table 2:** Pre- and postoperative measurements of the FTA, JCLA, and MPTA

		Preop	Postop 0 day	Postop 2 days	Postop 1 month	Postop 12 months
		Standing	Supine	Supine	Standing	Standing
FTA	OWHTO	182.5 ± 2.0	170.5 ± 2.1 ^b^	168.6 ± 2.2^b, c^	168.6 ± 2.3^b, c^	169.1 ± 1.8^b, c^
	CWHTO	187.6 ± 3.4 ^a^	168.7 ± 2.4 ^a, b^	168.1 ± 2.8^b^	168.7 ± 4.5^b^	168.6 ± 4.6^b^
JCLA	OWHTO	4.8 ± 1.8	4.2 ± 1.9 ^b^	2.2 ± 1.8^b, c^	2.6 ± 1.7^b, c^	2.7 ± 1.6^b, c^
	CWHTO	7.1 ± 3.2 ^a^	4.1 ± 2.4 ^b^	3.4 ± 2.5^a, b^	3.9 ± 2.3^a, b^	4.2 ± 2.6^a, b^
MPTA	OWHTO	83.6 ± 2.6	N/A	N/A	96.2 ± 2.8^b^	95.7 ± 2.7^b^
	CWHTO	82.2 ± 3.3 ^a^	N/A	N/A	97.8 ± 4.1^a, b^	97.6 ± 4.2^a, b^

*FTA* femorotibial angle, *JLCA* joint line convergence angle, *MPTA* medial proximal tibial angle

^a^*P* < 0.05 vs OWHTO

^b^*P* < 0.05 vs preop

^c^*P* < 0.05 vs postop 0 day

The ICCs for inter-rater and intra-rater reliabilities were all > 0.8, ranging from 0.89 to 0.98 for all radiological measurements, indicating good reliability.

### Difference in timing of the change of the JLCA

**Fig. 1:**
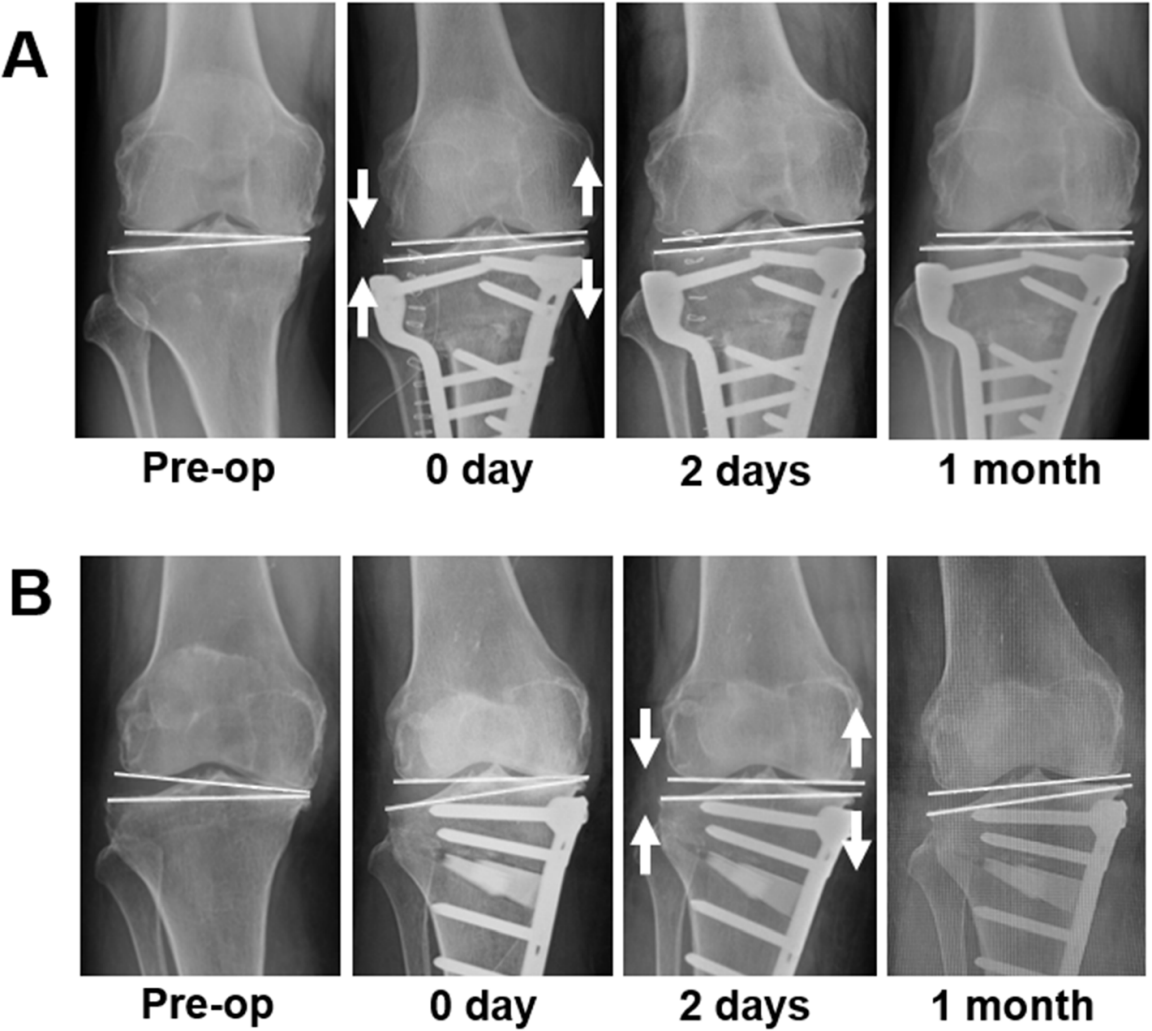
Perioperative change of the joint line convergence angle (JLCA) preoperatively (Pre-op), immediately under general anesthesia postoperatively (0 day), 2 days, and 1 month postoperatively in closed wedge high tibial osteotomy (CWHTO; **a**) and opening wedge high tibial osteotomy (OWHTO; **b**). Decrease of the JLCA by opening of the medial joint space and closing of the lateral joint space is shown at 0 day in CWHTO (**a**) and at 2 days in OWHTO (**b**), and it is maintained thereafter

The means and distributions of the degrees of JLCA change are summarized in Table 3. From preoperative to postoperative 0 day, the mean decrease of the JLCA was significantly greater in CWHTO than in OWHTO (*P* < 0.001), and decrease of the JLCA by ≥ 2° was observed in 63 knees (74%) in CWHTO and 25 knees (38%) in OWHTO (*P* < 0.001). From postoperative 0 day to 2 days, the mean decrease of the JLCA was significantly greater in OWHTO than in CWHTO (*P* < 0.001), and decrease of the JLCA by ≥ 2° was observed in 23 knees (27%) in CWHTO and 54 knees (64%) in OWHTO (*P* < 0.001). From postoperative 2 days to 1 month, and from postoperative 1 to 12 months, the mean and distribution of the JLCA change were not significantly different between the two HTO procedures. These results suggested that a greater decrease of the JLCA occurred intraoperatively in CWHTO, whereas it was delayed postoperatively in OWHTO (Fig. 1).

**Table 3:** Mean and distribution of degrees of change of the joint line convergence angle (JLCA)

		Preop to postop 0 day		Postop 0 to 2 days		Postop 2 days to 1 month		Postop 1 to 12 months	
		OWHTO	CWHTO	OWHTO	CWHTO	OWHTO	CWHTO	OWHTO	CWHTO
Mean		− 0.6 ± 1.5	− 3.1 ± 2.8	− 2.0 ± 1.4	− 0.7 ± 1.5	0.1 ± 1.3	0.4 ± 2.1	0.1 ± 1.2	0.4 ± 1.5
		*P* < 0.001		*P* < 0.001		*P* = 0.55		*P* = 0.36	
Number	≤− 2°	25	63	54	23	8	10	5	4
	− 1°	22	7	17	25	12	13	12	13
	0°	21	7	12	19	27	18	39	31
	1°	12	5	2	12	21	21	19	20
	≥ 2°	5	3	0	6	17	23	10	17
		*P* < 0.001		*P* < 0.001		*P* = 0.56		*P* = 0.57	

Note that radiographs were taken in the standing position at preoperative and postoperative 1 and 12 months, and in the supine position at postoperative 0 and 2 days

### Factors affecting early postoperative change of the JLCA

The correlation analysis for preoperative factors affecting the change of the JLCA from preoperative to postoperative 1 month is summarized in Table 4. A negative correlation was found between change of the JLCA and the correction angle (*P* < 0.001), preoperative standing FTA (*P* = 0.002), and preoperative standing JLCA (*P* < 0.001) in OWHTO, and between change of the JLCA and preoperative standing FTA (*P* < 0.001) and preoperative standing JLCA (*P* < 0.001) in CWHTO. Furthermore, multiple regression analysis for preoperative factors affecting change of the JLCA from preoperative to postoperative 1 month was performed (Table 5). The correction angle in OWHTO (*P* = 0.001) and the preoperative standing JLCA in OWHTO (*P* < 0.001) and CWHTO (*P* < 0.001) were related to early postoperative change of the JLCA.

**Table 4:** Correlation analysis for preoperative factors affecting change of the standing JLCA from preoperative to postoperative 1 month

Preoperative factors	OWHTO		CWHTO	
	*r*	*P* value	*r*	*P* value
Age	0.069	0.522	− 0.033	0.763
Body mass index	0.060	0.583	− 0.080	0.465
Correction angle	− 0.404	< 0.001	− 0.158	0.149
Preoperative standing FTA	− 0.337	0.002	− 0.524	< 0.001
Preoperative standing JLCA	− 0.549	< 0.001	− 0.708	< 0.001
Preoperative MPTA	0.021	0.845	0.045	0.680

*FTA* femorotibial angle, *JLCA* joint line convergence angle, *MPTA* medial proximal tibial angle

**Table 5:** Multiple regression analysis for preoperative factors affecting change of the standing JLCA from preoperative to postoperative 1 month

Independent variable	OWHTO				CWHTO			
	Unstandardized coefficient		Standardized coefficient	*P*	Unstandardized coefficient		Standardized coefficient	*P*
	Β	SE	β		Β	SE	β	
Age	0.014	0.022	0.069	0.522	− 0.001	0.019	− 0.002	0.982
Sex	0.665	0.353	0.170	0.063	0.566	0.433	0.103	0.195
Body mass index	0.068	0.047	0.131	0.148	0.001	0.063	0.001	0.987
Correction angle	− 0.211	0.062	− 0.352	0.001	0.036	0.061	0.050	0.563
Preoperative standing FTA	0.041	0.102	0.050	0.690	− 0.159	0.089	− 0.225	0.078
Preoperative standing JLCA	− 0.504	0.100	− 0.552	< 0.001	− 0.587	0.087	− 0.716	< 0.001
Preoperative MPTA	− 0.084	0.080	− 0.133	0.296	0.149	0.088	0.185	0.095

*FTA* femorotibial angle, *JLCA* joint line convergence angle, *MPTA* medial proximal tibial angle, *SE* standard error

## Discussion

This study focused on the discrepancy in radiographic assessment between the intraoperative supine position under general anesthesia and the postoperative standing position in OWHTO and CWHTO. The most important finding of the present study was that the JLCA was decreased postoperatively in both HTO procedures, but its timing was different. In addition, the factor affecting early postoperative change of the JLCA was the preoperative standing JLCA in both HTO procedures.

One of the recent topics in HTO is that lower limb alignment is affected by soft tissue balance, as well as the bony correction angle. Intraoperative confirmation of target alignment is carried out using supine fluoroscopy under anesthesia. However, the accurate limb alignment obtained intraoperatively is often lost in the postoperative standing radiograph under awake conditions [5]. A previous study demonstrated that factors related to > 10 mm discrepancy in measurement of mechanical axis deviation (MAD) between the preoperative standing radiograph and intraoperative supine fluoroscopy were limbs with MAD > 2 cm and those with a JLCA > 3° [17]. A similar study showed that the mean difference in MAD following OWHTO between intraoperative fluoroscopy and the postoperative standing radiograph was approximately 6%, and a greater discrepancy between them was significantly associated with higher BMI and JLCA values [18]. Thus, understanding the extent and conditions in the change of the JLCA is important for obtaining accurate limb alignment after HTO.

**Fig. 2:**
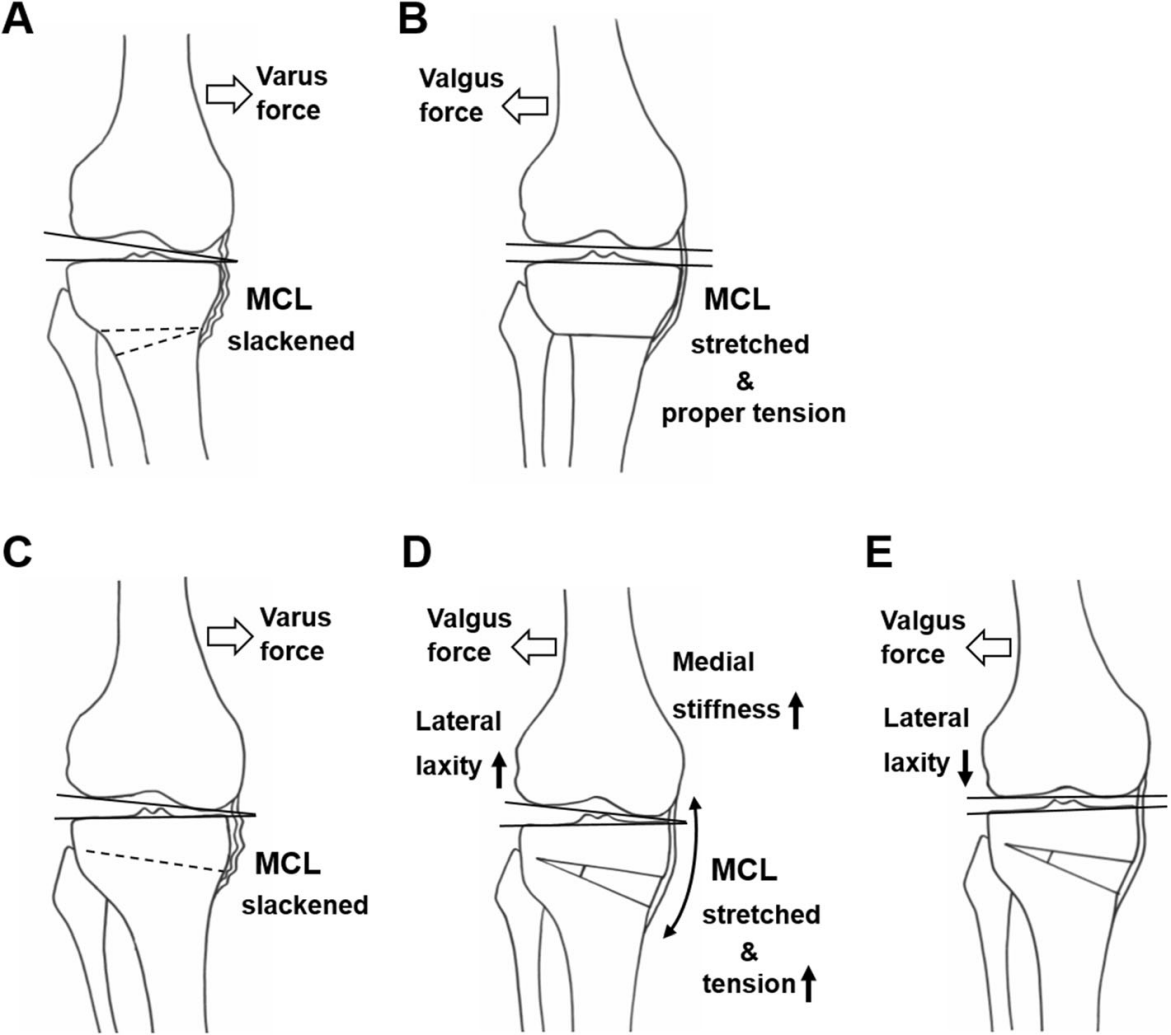
Difference between closed wedge high tibial osteotomy (CWHTO; **a**, **b**) and opening wedge high tibial osteotomy (OWHTO; **c**–**e**) in postoperative change of joint line convergence angle. Schematic descriptions show preoperative (**a**) and postoperative (**b**) soft tissue balance in CWHTO, and preoperative (**c**) and postoperative soft tissue balance with (**d**) and without (**e**) general anesthesia in OWHTO

It is assumed that the effects of surgical procedures on soft tissue balance differ between CWHTO and OWHTO. OWHTO increases ligament tension since osteotomy is performed proximal of the distal attachment of the MCL. In contrast, osteotomy site does not affect collateral ligament in CWHTO. Medial hyperlaxity is observed in OA knee with varus deformity due to loss of the medial joint space [19, 20] (Fig. 2a, c). In CWHTO, medial soft tissue with laxity is stretched to the proper tension by valgus correction, and the JLCA is decreased with opening of the medial joint space (Fig. 2b). Postoperative change of the JLCA occurs immediately after valgus correction under general anesthesia, and it is maintained later without general anesthesia. In contrast, tension of the medial soft tissue is excessively increased by opening of the medial structure at the osteotomy site, and medial laxity is not increased regardless of MCL release in OWHTO [21, 22] (Fig. 2d). Since lateral laxity is increased under general anesthesia [23], the JLCA is not decreased intraoperatively in OWHTO. The intraoperative increase of lateral laxity relative to the medial side under general anesthesia is thought to be reversed postoperatively in the awake condition, and the JLCA is decreased (Fig. 2e). This decreased JLCA may be maintained by lateral shift of the weight-bearing line [5].

This study demonstrated that the JLCA was decreased by valgus correction in both HTO procedures. Therefore, ideally, the amount of correction in the soft tissue part, as well as bony correction, needs to be included in preoperative planning. However, the extent of its effect varies widely in each individual, and it is difficult to determine the exact amount of soft tissue correction preoperatively. In the clinical setting, most surgeons might confirm intraoperative alignment to avoid under or over correction. As a point to note, they need to take into account the difference of timing in the change of the JLCA between the two HTO procedures. Intraoperative confirmation of limb alignment after osteotomy might be directly helpful in CWHTO, since the decrease of the JLCA occurred mainly intraoperatively, and the intraoperative FTA tended to be unchanged postoperatively. In contrast, it is unlikely that intraoperatively confirmed limb alignment is consistent with postoperatively confirmed alignment in OWHTO, since the decrease of the JLCA was relatively small intraoperatively, and it mainly occurred later. Therefore, it should be noted that the actual alignment after OWHTO is more likely to be overcorrected than the intraoperatively confirmed alignment in cases with a large preoperative JLCA.

This study has several limitations. First, the follow-up time of 1 month was short, since current series assessed the early change of knee alignment. Second, the present series did not include data for clinical outcomes, since that was not the primary aim. Third, the indication for either HTO technique was determined preoperatively according to the correction angle.

## Conclusions

Early postoperative change of the JLCA was found in both OWHTO and CWHTO, but its timing differed between the two HTO procedures. A significant decrease of the JLCA occurred immediately after osteotomy under anesthesia in CWHTO, whereas it occurred several days later in OWHTO. It is important to note that intraoperative accurate limb alignment is likely to be lost after OWHTO in cases with a large preoperative JLCA.

## Data Availability

The data and materials used and/or analyzed during the current study are not publicly available but available from the corresponding author on reasonable request.

## Abbreviations

OA: Osteoarthritis
OWHTO: Opening wedge high tibial osteotomy
CWHTO: Closed wedge high tibial osteotomy
FTA: Femorotibial angle
JLCA: Joint line convergence angle
MPTA: Medial proximal tibial angle
